# Assessing the Alignment Between Naturally Adaptive Grain Crop Planting Patterns and Staple Food Security in China

**DOI:** 10.3390/foods14223870

**Published:** 2025-11-12

**Authors:** Zonghan Zhang, Qiuchen Hong, Yihang Sun, Jinmin Hao, Dong Ai

**Affiliations:** College of Land Science and Technology, China Agricultural University, Beijing 100193, China; zzhan@cau.edu.cn (Z.Z.); simonhong@cau.edu.cn (Q.H.); bs20223211062@cau.edu.cn (Y.S.); jmhao@cau.edu.cn (J.H.)

**Keywords:** grain crops, natural suitability, nutrient supply, sustainable food production

## Abstract

Climate change and socio-economic transformation increasingly challenge the stability of China’s food supply. This study aims to optimize grain crop layouts by integrating natural suitability and nutritional supply within a unified analytical framework. Using the MaxEnt model incorporating bioclimatic, topographic, and soil variables, we simulated the natural suitability of major grain crops and compared it with actual planting patterns based on the SPAM dataset. Results revealed substantial spatial discrepancies between actual and suitable distributions, with national planting diversity index increasing by 26.42% (from 0.53 to 0.67) under suitable conditions. Wheat and maize are most suited to northern China, rice and tuber crops to southern regions, while soybean performs optimally in the northeast. Nutrient supply potential also improved substantially under the suitable scenario, with energy, protein, fat, and carbohydrate increasing by 56.9 × 10^8^ KJ, 77.2 × 10^6^ g, 23.3 × 10^6^ g, and 48.6 × 10^6^ g per million people, respectively. Among alternative structures, maize-soybean and maize-based planting structures better aligned with both natural adaptability and nutritional balance (e.g., in Inner Mongolia and Heilongjiang), whereas rice-based structure showed weaker correspondence (e.g., in Shanghai). These findings demonstrate that naturally adaptive optimization can enhance both environmental compatibility and nutritional adequacy, providing scientific guidance for developing climate-resilient and nutrition-oriented crop layout strategies in China.

## 1. Introduction

Global and national food security depend on maintaining a sustainable balance between food supply and demand [1,2]. However, escalating geopolitical tensions, growing dietary affluence, and rising production costs have destabilized the equilibrium between food availability and consumption [3,4,5]. This instability poses major challenges to achieving the United Nations Sustainable Development Goal (SDG 2) of ending hunger and ensuring food security by 2030 [6,7]. Socioeconomic development and rapid dietary transitions have further intensified food demand pressures [8]. Strengthening sustainable food production capacity is therefore essential to meeting future demands while advancing the SDGs [9,10]. Furthermore, agricultural diversification is pivotal in promoting dietary diversity [11,12] and improving nutritional health [13]. Hence, adapting agricultural production systems is imperative not only to mitigate food shortages, but also to ensure balanced, nutritious and sustainable dietary structures [14]. In recent years, China’s food security landscape has entered a new stage. With the introduction of the new food security concept emphasizing “basic self-sufficiency in grain and absolute security of staple food” and the strategy to “store grain in the ground and in technology”, China’s grain production capacity has significantly improved [15,16]. The Central Government’s No. 1 Document (2016) formally proposed the “All-encompassing Approach to Food”, which extends the scope of food security from ensuring food quality to encompassing the nutritional well-being of the population [17]. More recently, the “New Round of Action Programme for Enhancing 100 Billion Catty Grain Production Capacity (2024–2030)” underscored the need to evaluate the food supply-demand balance and regional resource endowment when optimizing grain crop layouts. This strategic framework emphasizes a transition from quantity-oriented to quality- and nutrition-oriented food security, placing higher demands on sustainable agricultural systems [18]. As China seeks to integrate food security with nutritional security, optimizing grain planting structures must consider both resource efficiency and population nutrition needs [19,20,21].

Meanwhile, the challenge of climate change adds another layer of complexity to food security. Building a climate-resilient agricultural system has become a key measure for the sustainable transformation of China’s agri-food system [22,23,24,25]. Enhancing climate resilience in agriculture is increasingly recognized as essential for helping farmers adapt to climate change [26,27]. As crop cultivation expands into regions previously deemed unsuitable, developing climate-resilient varieties is becoming critical for maintaining food security [28]. Beyond selecting drought- or heat-tolerant species, adaptive capacity also depends on effective agroecosystem management, including the use of high-yield and resistant cultivars, optimized sowing schedules, improved irrigation systems, and conservation practices such as crop rotation and no-tillage [29,30,31]. Collectively, these measures mitigate environmental stress, enhance soil health, and strengthen the overall resilience of agricultural systems. National strategies such as the Climate Change Adaptation Strategy 2035 aim to enhance agricultural resilience through policy guidance and technological innovation [32,33]. Studies have shown that climatic, edaphic, and topographic factors jointly determine crop suitability, yield potential, and spatial distribution [34,35,36]. These factors interact to define agro-ecological suitability thresholds, production potential, and the spatial distribution characteristics of different grain crops. Therefore, assessing natural suitability—the extent to which environmental conditions support crop growth—is essential for guiding adaptive agricultural planning [37]. The naturally adaptive planting strategy optimizes regional resource utilization, ensures high and stable yields, and mitigates the environmental footprint of production [38,39]. However, most existing research focuses on the environmental or biophysical dimensions of crop layout optimization, often overlooking the nutritional outcomes of such spatial adjustments.

China’s grain production pattern is currently undergoing a distinct “south retreat and north advance” shift, driven by both natural environmental changes and socio-economic drivers [34,40]. This transition has intensified spatial fragmentation and regional heterogeneity in cropland use [41,42]. Previous studies primarily addressed food security from the perspective of yield stability and environmental sustainability, yet few have integrated nutritional considerations into spatial optimization frameworks [43,44,45]. As a result, the linkage between natural suitability and nutrient supply potential remains insufficiently explored. Accordingly, this study aims to: (a) evaluate the natural suitability of major grain crops using the MaxEnt model and identify the dominant environmental drivers; (b) compare actual and potential planting patterns to quantify planting diversity, production, and nutrient supply under both conditions; and (c) develop region-specific strategies for optimizing grain crop layouts to enhance productivity, nutritional security, and sustainability. This research establishes a comprehensive framework that integrates natural suitability with nutritional benefits, offering theoretical and practical insights for developing climate-resilient and nutrition-sensitive food systems in China.

## 2. Materials and Methods

### 2.1. Data Collection

#### 2.1.1. Grain Crop Sample Data

The grain crop sample points were sourced from the Spatial Production Allocation Model (SPAM, https://mapspam.info/) for the year 2020. SPAM provides data on harvested area, actual area, yield and total production at a 5 arc-minute grid resolution for 42 crops worldwide. In this study, planting distribution data for wheat, maize, rice, soybean, potato, sweet potato and cassava in SPAM2020 (SPAM2020 V1.0, https://doi.org/10.7910/DVN/SWPENT, accessed on 9 April 2024) were extracted from grids representing the actual cultivated area. Each grid value represents the proportion of grain crops cultivated within that specific grid [46].

#### 2.1.2. Environmental Data

Climatic conditions and soil quality are primary environmental determinants influencing grain crop production, while topography further influences grain crops growth [47,48]. In this study, 19 bioclimatic variables, 4 landform variables, and 19 soil variables were incorporated into the MaxEnt model to determine the natural, long-term habitat suitability (ecological niche) of a given crop under stable conditions. The bioclimatic variables represent 1970–2000 averages with a resolution of 30 s (~1 km^2^) from WorldClim version 2, obtained from the WorldClim database (https://www.worldclim.org/). Landform variables were derived from the 30 m resolution SRTM dataset, while soil variables were obtained from the Harmonized World Soil Database (HWSD) (https://www.fao.org/soils-portal/soil-survey/soil-maps-and-databases/harmonized-world-soil-database-v12/en/, accessed on 7 March 2012) at a 1 km resolution. The Soil Map of China was compiled using soil data from the Second National Soil Survey (1995) [49]. The environmental variables were incorporated into the MaxEnt model, with their descriptions presented in Table S1, totaling 42 variables.

### 2.2. Data Preprocessing

#### 2.2.1. Identification of Grain Crops’ Planting Sites

**Figure 1:**
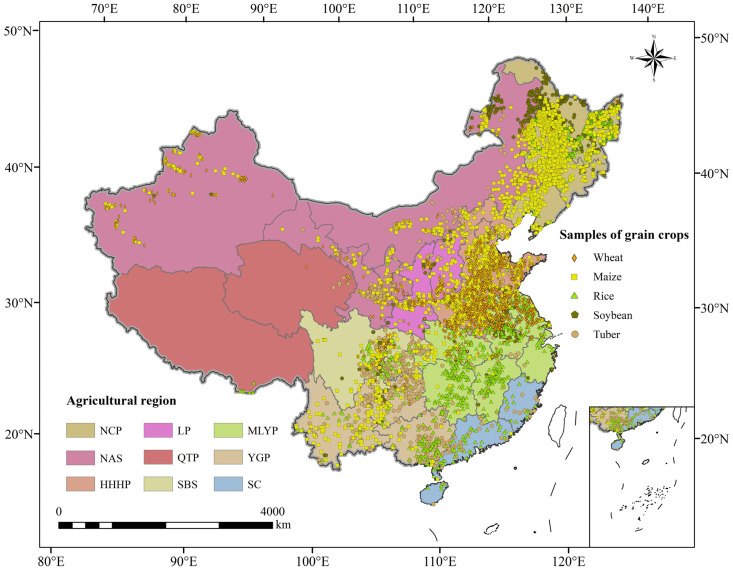
Study area and the distribution of grain crops’ planting samples.

This study uses provinces as the primary research scale and defines agricultural regions as the fundamental unit for guiding and managing agricultural production. The agricultural regions considered in this study include the Northeast China Plain (NCP), Northern Arid and Semiarid Region (NAS), Huang-Huai-Hai Plain (HHHP), Loess Plateau (LP), Qinghai–Tibet Plateau (QTP), Sichuan Basin and Surrounding Region (SBS), Middle-Lower Yangtze Plain (MLYP), Yunnan-Guizhou Plateau (YGP) and Southern China (SC) (Figure 1).

Crop suitability evaluation begins with the identification of planting sites. In this study, SPAM data representing the proportional area of each crop within 1 km^2^ grid units were used to classify target planting locations into six frequency zones: Category 1 (0.8–1), Category 2 (0.6–0.8), Category 3 (0.4–0.6), Category 4 (0.2–0.4), Category 5 (0.05–0.2), and Category 6 (0–0.05). This classification enables the MaxEnt model to be trained mainly on the most representative and stable locations, thereby improving the reliability of suitability predictions. A stratified sampling strategy was subsequently applied, with sample points extracted in varying proportions from each category: 90% from Category 1, 70% from Category 2, 50% from Category 3, 30% from Category 4, and 10% from Category 5. Category 6 was excluded due to its negligible planting frequency. Finally, samples from Categories 1 to 5 were merged into a consolidated dataset to generate planting sample layers for wheat, maize, rice, soybean, and tubers (including potato, sweet potato, and cassava) (Figure S1).

#### 2.2.2. Selection of Environment Variables

This study involves 42 environmental variables, which may exhibit significant spatial autocorrelation within each group. To prevent overfitting due to multicollinearity among environmental factors, this study initially inputted all variables into the MaxEnt model, obtained preliminary model simulation results and assessed the contribution of each environmental factor using Dojackknife analysis [50,51]. Subsequently, variables with contribution rates below 1% were excluded, and Spearman rank correlation analysis was conducted on the remaining environmental factors using ENMtools (v.1.1.5). Based on the correlation results (Tables S2–S15), a subset of environmental variables with a correlation coefficient of ≥|0.9| was selected, while those with lower contributions to model prediction were excluded based on a comparative assessment of both variables’ contributions. Ultimately, the most influential environmental variable was identified and selected as the input variable for suitability modeling of each grain crop. The final MaxEnt model inputs included 12 environmental variables for wheat, 10 for maize and rice, 11 for soybean and 13, 8, and 8 for potato, sweet potato, and cassava, respectively.

### 2.3. Application of the MaxEnt Model

The geographical distribution data for wheat, maize, rice, soybean, potato, sweet potato, cassava, and environmental variables were entered into the MaxEnt model for predictive modeling. In the model, the random test percentage represents the proportion of test sets randomly selected from the sample data. The regularization multiplier is used to control model complexity and prevent overfitting, while the replicate parameter determines the number of times the model is randomly tested. The model parameters were set as follows: 25% of the distribution points were designated as the test set, and 75% as the training set. Cross-validation was applied, where the species distribution data were randomly partitioned into 10 subsets. In each iteration, one subset was used as the test set while the remaining nine served as the training set. This process was repeated 10 times to maximize data utilization. The maximum number of iterations was set to 500, the maximum number of background points to 10,000, and the number of iterations was set to 10, while all other parameters were kept at their default values. The final ASCII output file represented the average results from the 10 iterations. The resulting file was imported into ArcGIS 10.8 and processed using the reclassification function in the Spatial Analysis tool. Crop suitability was classified into six levels using the natural break (Jenks) classification method: Perfectly suitable (0.8–1), Highly suitable (0.6–0.8), Moderately suitable (0.4–0.6), Lowly suitable (0.2–0.4), Marginal suitable (0.05–0.2), and Unsuitable (0–0.05). Thus, the suitability distribution of each food crop in China was generated.

This study considered two distinct conditions: actual and suitable. The actual condition represents the observed distribution of major grain crops based on the SPAM 2020 dataset, which reflects the combined effects of environmental suitability and socio-economic drivers such as agricultural policy, land management, and market accessibility. The suitable condition, in contrast, refers to the potential natural suitability of crops estimated by the MaxEnt model. This suitability is determined solely by environmental factors, including bioclimatic, topographic, and soil variables, without accounting for socio-economic constraints. The comparison between actual and suitable conditions was performed through spatial overlay analysis and divisional statistics (by agricultural regions and provinces). Specifically, actual planting distributions were overlaid with the MaxEnt-derived suitability maps to evaluate spatial layout and structure, quantify the differences in planting diversity, and assess potential improvements in production and nutrient supply under naturally suitable conditions.

### 2.4. Measurement of Grain Crops’ Diversity

In this study, the Simpson Diversity Index (SDI) from ecology was applied to assess the cultivation diversity of regional grain crops [52,53]. This study primarily focused on five types of grain crops: wheat, rice, maize, soybean, and tuber crops (including potato, sweet potato, and cassava). The index presents the likelihood that two randomly selected individuals from a community belong to the same species, thus capturing both the uniformity of crop distribution and the overall diversity of cultivated crop types within a given region. Food crop diversity at the provincial scale was calculated using Equation (1) as follows:

(1) $$SDI=1-\sum_{i=1}^{S}P_{i}^{2}$$

In the formula, *SDI* indicates the diversity index of regional grain crop planting, and its value range is [0, 1), the larger the value, the higher the degree of diversity of regional planting types (i.e., the more types of crop planting and the more it tends to be uniformly distributed); *S* denotes the number of types of grain crops in the study period; $P_{i}$ denotes the regional share of the area planted with grain crops of type *i*.

### 2.5. Calculation of Grain Crops’ Nutrient

The average dietary nutrient demand of adult men aged 18 years and above with light physical activity was used as the reference intake standard, and the unit of calculation is 100 g [54]. Refer to the food energy and nutrient indicators for protein, fat and carbohydrate (CHO) in the Chinese Food Composition Table (Sixth Edition). The consistency of the nutrient content of different grain crops was measured by means of the food nutrient transformation model (Table 1). The food nutrient transformation model was expressed as Equation (2):

(2) $$NRQ_{ij}=RQ_{i}\times e_{ij}\times \frac{\left(1-w_{i}\right)}{10}$$

*NRQ_ij_* is the nutrient content of category *j* for grain crop *i*, where *i* = 1, 2…, 5, represents wheat, maize, rice, soybean, tuber, and *j* = 1, …, 4, represents energy, protein, fat, and CHO, respectively; *RQ_i_* is the total yield (kg) of the food crop *i*; *e_ij_* is the nutrient content of category *j* contained in per 100 g of grain crop *i*; *w_i_* is the water content per 100 g of grain crop *i*.

**Table 1:** Energy and nutrient contents of food crops (per 100 g edible part).

Nutritional Index	Wheat	Rice	Maize	Soybean	Tuber	Potato	Sweet Potato	Cassava
Energy (KJ/100 g)	1416	1457	1453	1631	367	343	260	498
Protein (g/100 g)	11.9	8.7	7.9	35.0	1.8	2.6	0.7	2.1
Fat (g/100 g)	1.3	3.8	0.9	16.0	0.2	0.2	0.2	0.3
CHO (g/100 g)	75.2	73.0	77.2	34.2	20.3	17.8	15.3	27.8
Edible (per cent)	100	100	100	100	94	94	90	99
Moisture content (g/100 g)	10.0	13.2	13.3	10.2	77.0	78.6	83.4	69.0

In order to measure the use value of various types of food in a uniform way, this study adopted the method of calculating food equivalent values [55]. The calculation of the grain equivalent was updated based on the food composition of Chinese Food Composition Table (6th edition), see Equation Group (3) to (6) for details. Rice was used as the reference standard food, and wheat, maize, soybean, and tuber were converted into rice equivalents according to their corresponding food equivalent coefficients (Table S16).

(3) $$FEU=H\times C_{H}+P\times C_{P}$$

(4) $$C_{H}=\frac{0.9}{H}\;,\;C_{P}=\frac{0.1}{P}$$

(5) $$FEU=\frac{H}{1614}+\frac{P}{79}$$

(6) $$FEU\prime =FEU\times \frac{E\times \left(100-W\right)}{E_{s}\times \left(100-W_{s}\right)}$$

where *FEU* is food equivalent, *H* is food energy (KJ/100 g), *CH* is the energy coefficient, *ES* is the standard edible portion (%), *E* is the actual edible portion (%), *P* is the protein content (g/100 g), *CP* is the protein coefficient, *WS* is the standard water content (g/100 g), *W* is the actual water content (g/100 g), and *FEU′* are corrected food equivalent.

## 3. Results

### 3.1. Suitable Planting Pattern of Grain Crop

#### 3.1.1. Model Performance

The average Area Under the Curve (AUC) values for the seven grain crops across 10 runs were 0.9259, 0.8534, 0.9177, 0.9354, 0.9370, 0.9627, and 0.9932 for wheat, maize, rice, soybean, potato, sweet potato, and cassava, respectively (Table 2). The prediction results indicated that the MaxEnt model demonstrated stability and high repeatability in selecting occurrence points for these seven grain crops (Figure S3). These findings suggested that the model offered reliable prediction for garin crop planting suitability in China.

#### 3.1.2. Influence of Environmental Variables on Planting Suitability Range

**Figure 2:**
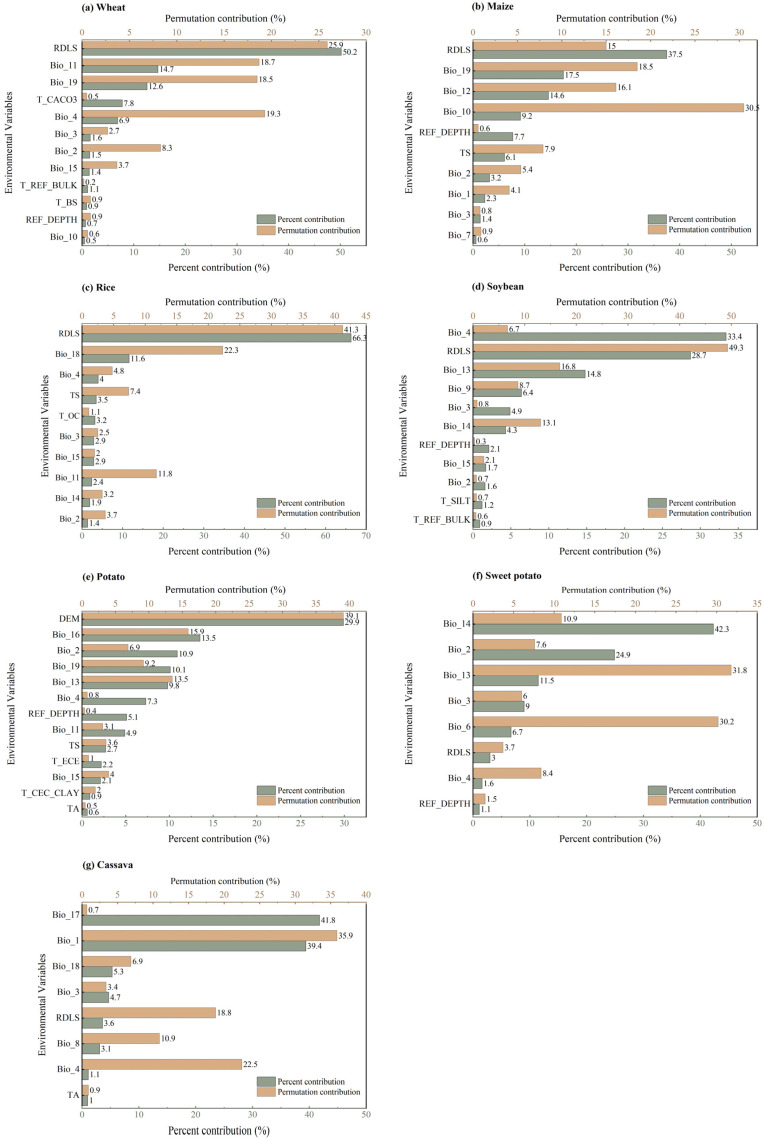
Percent contribution and permutation contribution of major environmental variables to the planting suitability evaluation of wheat (**a**), maize (**b**), rice (**c**), soybean (**d**), potato (**e**), sweet potato (**f**), and cassava (**g**) crops.

The relative importance of environmental factors exhibited variation across the grain crops analyzed (Detailed information can be found in Figure 2, the full names of factors can be seen in Table S1). For wheat, topographic factors, specifically the relief amplitude (RDLS), accounting for 50.2% of the explained variation in planting suitability. This was followed by the precipitation of the coldest quarter precipitation (Bio_19) and the reference soil depth (REF_DEPTH). Similarly, the RDLS emerged as the most significant factor for both maize and rice, with substantial contributions of 37.5% and 66.3%, respectively. The secondary factors for maize were the Bio_19 and the annual precipitation (Bio_12), whereas for rice, the warmest quarter precipitation (Bio_18) and the coefficient of seasonal variation in temperature (Bio_4) are of secondary importance. For potato, altitude (DEM) was identified as the most critical factor, contributing 29.9%, followed by the precipitation in the driest quarter (Bio_16) and the average daily temperature difference (Bio_2). In contrast, the planting suitability of soybean, sweet potato, and cassava was primarily governed by climatic variables. For soybean, Bio_4 was the leading factor (33.4%), followed by RDLS and the precipitation in the wettest month (Bio_13). Similarly, sweet potato suitability was most affected by precipitation-related factors, with the precipitation in the driest month (Bio_14) contributing 42.3%. For cassava, the wettest quarterly precipitation (Bio_17) showed the highest contribution (41.8%).

In summary, topographic factors predominantly determined the planting suitability of wheat, maize, rice, and potato, whereas climatic factors, particularly temperature and precipitation, exerted greater influence on soybean, sweet potato, and cassava. These contrasts reflected differences in crop physiology and cultivation systems. Terrain attributes directly affect soil moisture retention, mechanization potential, and irrigation efficiency, thus shaping the suitability of cereal crops and potato grown on large, mechanized areas. In contrast, soybean, sweet potato, and cassava are more sensitive to hydrothermal regimes fluctuations during key growth stages can substantially alter their yield and quality.

### 3.2. Comparison of Actual and Suitable Planting Patterns

#### 3.2.1. Analysis of Grain Crops’ Planting Layout

**Figure 3:**
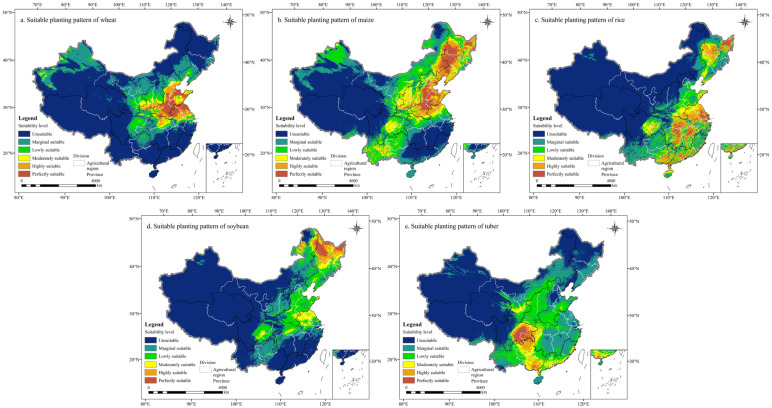
Suitable planting patterns of wheat (**a**), maize (**b**), rice (**c**), soybean (**d**), and tuber (**e**) in China.

The actual and suitable planting patterns of major grain crops in China were, respectively, illustrated in Figure S4 and Figure 3. Distinct spatial differentiation was evident across both patterns. For wheat and maize, the actual planting patterns were highly concentrated in regions that also coincided with areas of high modeled suitability, particularly the southern HHHP and LP (e.g., Henan, Anhui, Jiangsu, and the northern part of Shandong) for wheat, and the HHHP and NCP (e.g., Henan, Hebei, and the western part of Heilongjiang, Jilin, Liaoning) for maize. Meanwhile, the suitable planting range of rice was broader than its actual distribution, extending across the MLYP, SC, and NCP, with core optimal zones in the northeastern Heilongjiang and the Yangtze River Delta. Soybean and tuber crops exhibited more localized spatial patterns. Both the actual and highly suitable planting areas of soybean were concentrated in the NCP, with Heilongjiang as the core production zone. For tuber crops, while the main actual planting areas were located in Guizhou, Yunnan, and Guangxi provinces, the suitable range extended more broadly into the SBS region, particularly Sichuan and the eastern parts of Chongqing. In addition, the AUC values calculated in Section 3.1.1, together with the overlap between actual and suitable distributions, effectively confirmed the reliability of the MaxEnt model employed in this study. Therefore, the crop suitability patterns illustrated in Figure 3 can be regarded as the robust representation of natural suitability, while the remaining discrepancies between actual and modeled suitability primarily reflect socio-economic and policy-driven land-use adjustments.

**Figure 4:**
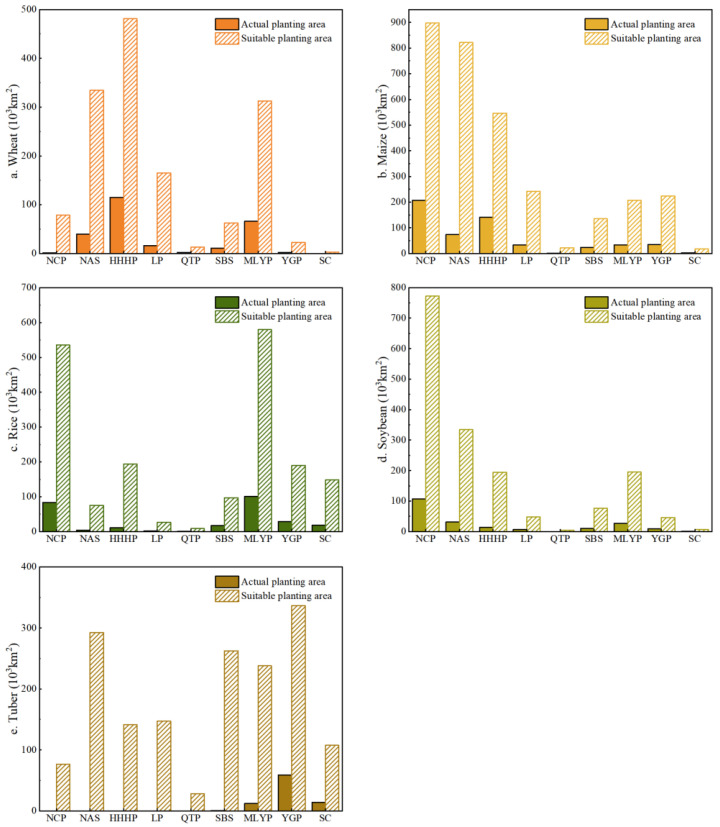
Comparison of actual and suitable planting area of wheat (**a**), maize (**b**), rice (**c**), soybean (**d**), and tuber (**e**) in the nine agricultural regions.

Comparing the actual planting area of grain crops with the suitable planting area, it was evident that the suitable planting area for China’s grain crops significantly exceeded the current actual planting area (Figure 4). This indicated a significant difference between the current distribution of grain crop cultivation and the distribution expected under optimal natural conditions. Meanwhile, the suitability of grain crops varies across regions, exhibiting distinct regional variations. Maize and wheat dominate in northern China, while tuber and rice are predominant in the south. This difference was also manifested in the spatial distribution of agricultural regions.

Specifically, as a key grain-producing region in China, the suitable planting area for maize, rice, and soybean in NCP was 897.46 × 10^3^ km^2^, 535.13 × 10^3^ km^2^, and 772.59 × 10^3^ km^2^, respectively, which was 4.3, 6.4, and 7.2 times the actual planting area. NAS exhibited a higher suitability for maize cultivation, reaching 821.64 × 10^3^ km^2^, while its actual planting area was relatively limited at 73.23 × 10^3^ km^2^. This highlighted the necessity for NAS to mitigate drought challenges and optimized cropping structures. HHHP’s suitable planting area for wheat and maize was 481.16 × 10^3^ km^2^ and 546.27 × 10^3^ km^2^, respectively, which was 4.2 and 3.9 times the actual planting area. LP demonstrates high suitable for maize and wheat cultivation, reaching 242.04 × 10^3^ km^2^ and 164.91 × 10^3^ km^2^ respectively, which was 7.3 and 10.4 times the actual planting area for these crops. QTP and SC had the highest suitability for tuber cultivation, with 28.28 × 10^3^ km^2^ and 262.60 × 10^3^ km^2^, respectively, exceeding the actual planting area of 28.21 × 10^3^ km^2^ and 262.09 × 10^3^ km^2^. MLYP had the most significant advantage in rice cultivation, with a suitable planting area of 579.43 × 10^3^ km^2^, which was 5.8 times the actual planting area. Although the actual rice planting area in MLYP was already substantial, there remained potential for expansion with improved environmental adaptability. YGP exhibited high suitability for tuber cultivation, with a suitable planting area of 336.58 × 10^3^ km^2^, which was 19.79 times the actual planting area. Meanwhile, the suitable planting areas for maize and rice in YGP were also significant, at 224.27 × 10^3^ km^2^ and 190.00 × 10^3^ km^2^, respectively. SC demonstrated high suitability for rice and tuber cultivation, with suitable planting areas reaching 148.28 × 10^3^ km^2^ and 107.57 × 10^3^ km^2^, respectively, which was 8.2 times and 7.0 times the actual planting area.

#### 3.2.2. Analysis of Grain Crops’ Planting Structure

**Figure 5:**
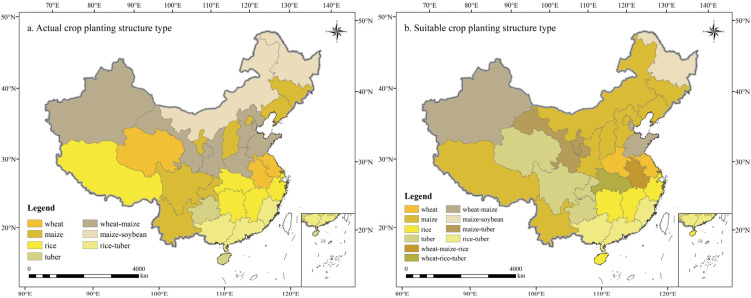
Comparison of planting structure types of grain crops under actual (**a**) and suitable (**b**) condition.

In this study, the crop planting structure refers to the combination of crop types planted within a specific region. Compared to the actual planting pattern, the diversity of grain crop planting structures across China increased under optimal suitability conditions, with the number of regional planting structures expanding from seven to ten. The newly emerged structures included maize-tuber, wheat-maize-rice, and wheat-rice-tuber (Figure 5). Maize remained the predominant crop type in China’s grain crop planting structure, with its suitable planting area significantly expanding under optimal conditions. Regionally, the planting structure in Heilongjiang within the NCP region remained maize-soybean, while Jilin and Liaoning continued to follow a maize-based structure. In NAS, the planting structure in Xinjiang remained wheat-maize, while Gansu and Ningxia shifted to a maize-tuber structure, and Inner Mongolia transitioned from maize-soybean to maize. In HHHP, the planting structure remained unchanged in Beijing, Tianjin and Shandong, while Hebei and Henan shifted from a wheat-maize structure to maize and wheat, respectively. In LP, the planting structure remained unchanged in Shanxi, while Shaanxi shifted from wheat-maize to maize. In QTP, Tibet transitioned from a rice-based to a maize-based structure, while Qinghai shifted from a wheat-based to a tuber-based structure. In SBS, both Sichuan and Chongqing transitioned from maize to tuber-based structure. The planting structure in MLYP was primarily rice-based but became more diversified under optimal conditions, shifting to a wheat-rice-tuber structure in Hubei and from wheat to wheat-rice-tuber in Anhui. The YGP’s planting structure remained consistent between actual and suitable conditions. In SC, only Hainan transitioned from a tuber-based to a rice-based structure, while Guangdong and Fujian had consistently maintained a rice-tuber structure.

### 3.3. Assessment of Differences Between Actual and Suitable Grain Crops’ Planting Effectiveness

#### 3.3.1. Analysis of Grain Crops’ Planting Diversity

**Figure 6:**
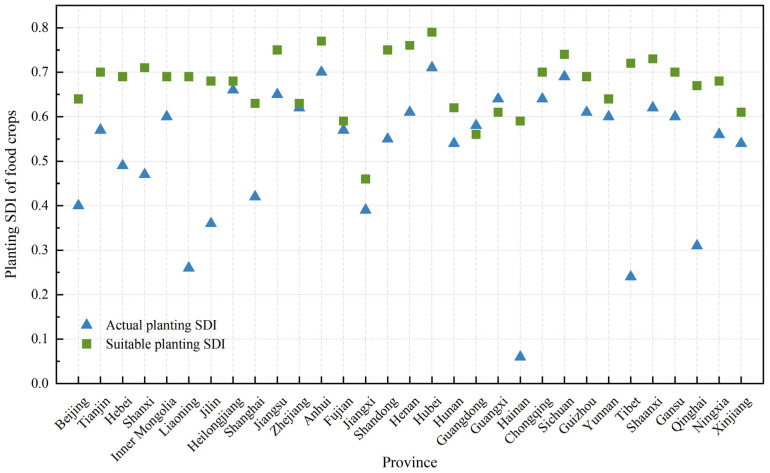
Comparison of actual and suitable planting SDI of grain crops by province.

The average SDI across China was 0.53 under the actual condition and 0.67 under the suitable condition, indicating a 26.42% increase in planting diversity due to natural suitability. In most provinces, SDI values under the suitable condition exceeded those under the actual condition, except in Guangdong and Guangxi, where higher diversity occurred in actual planting. Hubei exhibited the highest SDI under both actual and suitable conditions, reaching 0.71 and 0.76, respectively (Figure 6, Table S17). Under the suitable condition, the share of wheat, maize, and rice in Hubei declined, while tuber crops expanded markedly to 20.10% of the regional planting area. Hainan showed the largest SDI difference between conditions, with maize and rice proportions increasing by 20.43% and 57.79%, respectively, and tuber crops decreasing to 18.11%.

#### 3.3.2. Analysis of Grain Crops’ Production

China’s primary wheat-producing region was concentrated in HHHP, while maize was primarily cultivated in HHHP and NCP. Rice production was mainly centered in MLYP, with some expansion into SC. Soybean cultivation was primarily concentrated in NCP, with some presence in HHHP, while tuber cultivation was largely concentrated in SBS and SC.

**Figure 7:**
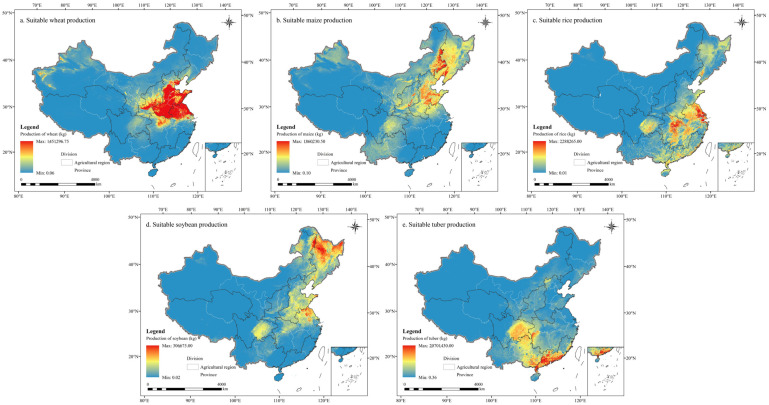
The distribution map of the production of wheat (**a**), maize (**b**), rice (**c**), soybean (**d**) and tuber (**e**) in China under the suitable condition.

China’s grain production under the suitable condition is generally higher than the actual production (Figure 7 and Figure S5 are, respectively, under the suitable and actual conditions). Under suitable condition, wheat production in Henan, Shandong and Hebei reached 16,239.59 × 10^7^ kg, 11,110.78 × 10^7^ kg, and 10,737.08 × 10^7^ kg, respectively, significantly exceeding actual production levels. Maize production in Inner Mongolia reaches 50,466.93 × 10^7^ kg, exceeding actual production by 45,925.57 × 10^7^ kg, while Heilongjiang recorded the second highest maize production at 32,473.03 × 10^7^ kg. Heilongjiang also had the highest production of both rice and soybean, with the largest disparity between actual and suitable production levels, reaching 9615.52 × 10^7^ kg and 21,890.21 × 10^7^ kg, respectively. Tuber production under the suitable condition was generally significantly higher than actual production levels, with relatively high production in Guangdong, Sichuan, and Guangxi, reaching 176,499.64 × 10^7^ kg, 151,813.35 × 10^7^ kg, and 136,345.40 × 10^7^ kg, respectively. Ningxia was the only region where the suitable production was lower than the actual production, with suitable rice production reaching 47.95 × 10^7^ kg, a decrease of 35.53 × 10^7^ kg compared to actual production.

#### 3.3.3. Analysis of Grain Crops’ Nutrient Supply

**Figure 8:**
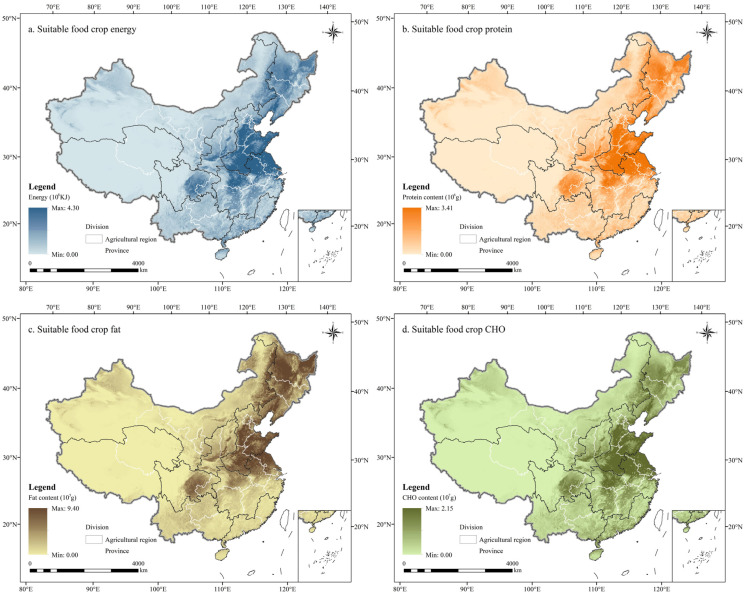
The distribution map of energy (**a**), protein (**b**), fat (**c**), and carbohydrate (**d**) contents provided by major grain crops in China under the suitable condition (rice as the standard food).

The spatial distribution of the energy, protein, fat and carbohydrate contents of grain crops was generally consistent, exhibiting a distribution pattern of “higher in the east and lower in the west” (Figure 8 and Figure S6 are, respectively, under the suitable and actual conditions). Specifically, regions with high energy and nutrient contents in grain crops were concentrated in HHHP and NCP, whereas lower values were observed in QTP.

**Figure 9:**
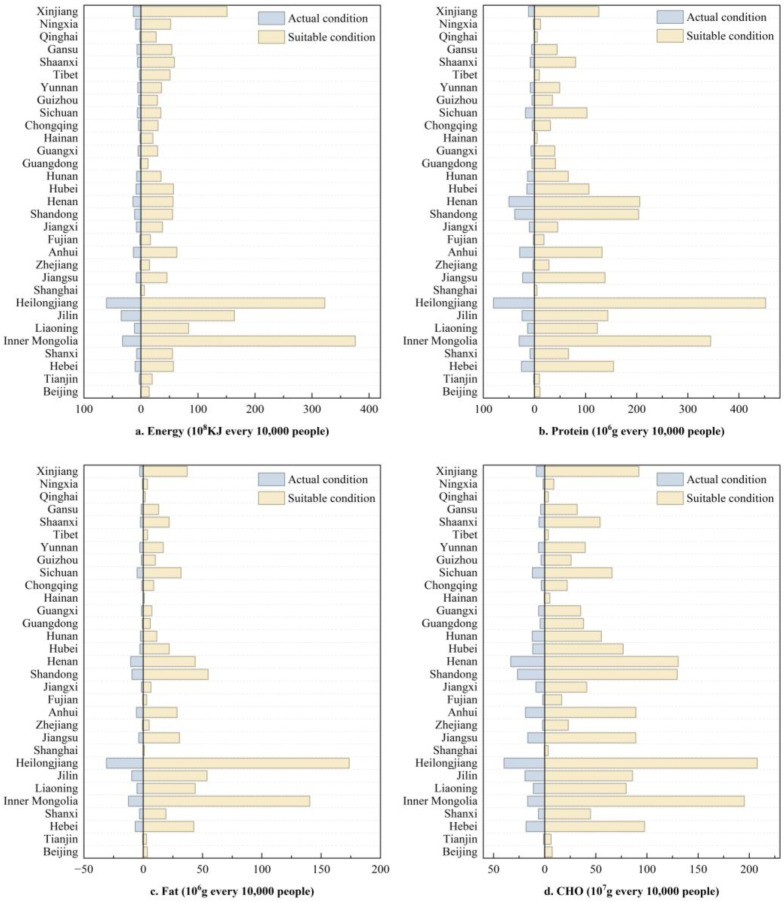
Comparison of per capita nutrient energy (**a**), protein (**b**), fat (**c**), and carbohydrate (**d**) contents of major grain crops under the actual and suitable conditions (rice as the standard food).

Under the suitable condition, Inner Mongolia had the highest per capita grain crop energy, reaching 375.76 × 10^8^ KJ per million people. Heilongjiang had the highest per capita nutrient content, with protein, fat and carbohydrate content reaching 452.17 × 10^6^ g, 173.59 × 10^6^ g and 207.81 × 10^7^ g per million people, respectively. Comparatively, Shanghai had the lowest per capita energy and nutrient content of grain crops under this condition, with per capita energy of only 6.11 × 10^8^ KJ per million people, and per capita protein, fat and carbohydrate content of 4.84 × 10^6^ g, 0.99 × 10^6^ g, and 3.53 × 10^7^ g per million people, respectively (Figure 9).

In Inner Mongolia, the differences in per capita energy and carbohydrate contents between the suitable and actual conditions were greater than in other provinces, reaching 343.51 × 10^8^ KJ and 178.55 × 10^7^ g per million people, respectively (Table S18). Heilongjiang exhibited the largest disparities in per capita protein and fat contents between both conditions, with values of 371.82 × 10^6^ g and 142.62 × 10^6^ g per million people, respectively. However, Shanghai had the smallest disparity in per capita energy and nutrient content between the two conditions, with values of 5.39 × 10^8^ KJ, 4.34 × 10^6^ g, 0.92 × 10^6^ g, and 3.10 × 10^7^ g per million people for energy, protein, fat, and carbohydrates, respectively.

In summary, with the development of local independent production and marketing, the potential for improving the energy and nutrient supply of grain crops is greater in Inner Mongolia and Heilongjiang, whereas the potential for improvement is more limited in Shanghai.

## 4. Discussion

### 4.1. Disparities Between Potential Suitability and Actual Planting Patterns

Global population growth will require agricultural production to increase by approximately 70% by the 2050s to meet basic food demands [20]. However, substantial cropland resources remain underutilized worldwide, and the inefficient spatial allocation of cropland continues to threaten sustainable development and food security [17,56]. Merely expanding production is insufficient to ensure human well-being; modern agriculture must simultaneously enhance yield, quality, and crop diversity to satisfy nutritional needs [57,58,59]. Thus, agricultural systems should balance productivity with environmental capacity while integrating nutrition and health considerations into sustainability frameworks [60,61,62].

The discrepancies observed between potential and actual planting patterns in this study reflected a universal challenge in aligning natural suitability with socio-economic realities. Similar findings have been reported globally. In South Africa, crops with high drought and heat tolerance remain underutilized despite favorable environmental conditions [63]. In Sub-Saharan Africa, sorghum suitability assessments highlight the tight linkage between environmental adaptation and nutrient provision in semi-arid regions [64]. And in Europe, political and fiscal regimes frequently influence crop-distribution patterns beyond natural site potential [65]. These global parallels underscore that land-use decisions are rarely determined by natural potential alone [66,67].

Our MaxEnt-derived potential distributions represent natural site suitability based on bioclimatic, topographic, and soil factors, whereas actual planting patterns reflect complex economic and institutional influences. Although actual planting areas largely contain within the potential ones (validating the model’s reliability), further optimization through structural adjustment, diversification, and varietal improvement remains essential to enhance regional production efficiency and resilience. Such integrated, nutrition-oriented, and climate-resilient strategies represent a crucial pathway toward sustainable food systems and improved nutritional security [68].

### 4.2. Policy Suggestions

Considering the regional variations in suitability and nutrient supply capacity of grain crops, the planting structure should be further optimized, and the regional planting layout should be rationally adjusted.

NCP is optimally suited for maize and soybean cultivation and highly suitable for rice production, aligning with the regional planting structure adjustment goal of “increasing the soybean planting area while stabilizing maize and rice planting area” [69]. Under natural adaptation conditions, the soybean planting area in the NCP reaches 772.59 × 10^3^ km^2^, significantly surpassing other regions. Additionally, as a region with high energy density and significant nutritional value in grain crop production, NCP contributes approximately 30% of the total national supply of energy and essential nutrients under optimal suitability conditions, demonstrating substantial growth potential compared to actual planting patterns. However, it is important to recognize that NCP still faces ecological and environmental challenges, including black soil degradation and loss, overexploitation of groundwater resources, and underutilization of surface runoff [70,71]. To address these issues, efforts should focus on enhancing the development of controlled hydro-junction projects for cropland irrigation and strengthening the protection and cultivation of fertile soil layers in black soil regions [72]. Furthermore, while ensuring the stability and security of regional grain production, it is crucial to effectively coordinate the relationship between grain production and regional economic development.

The crop energy and nutrient content of NAS under the suitable condition generally increase by 6.5% to 8.5% compared to the actual condition. This indicates that natural adaptive management measures enhance the energy and nutrient supply of regional grain crops. This is because the potential grain planting space (i.e., reserve cropland resources) in this region is subject to environmental constrains such as drought and water shortage, soil salinization, wind erosion and sand encroachment, and extreme low temperatures. These factors contribute to a fragile regional ecosystem, posing technical challenges and ecological risk associated with development and utilization [73]. Given these challenges, strategic water resource management has become a critical measure for enhancing cropland conditions and improving the production potential of existing cropland. Therefore, through the construction of water supply projects, the irrigation system can be effectively optimized, significantly enhancing the suitability of grain cultivation under natural conditions [74].

HHHP, LP and SBS are diverse grain-producing regions, all of which achieved an SDI exceeding 0.70. The advantages of regional crop diversification can be leveraged to integrate food and cash crops, thereby simultaneously enhancing benefits and nutritional value. In addition, HHHP and LP exhibit no difference between actual and suitable planting structures, which correspond to the maize-wheat structure and maize-based structure, respectively, whereas SBS has the potential to shift from a rice-based to a tuber-based structure. Among these regions, HHHP is the most suitable for wheat cultivation, with actual and suitable planting areas of 115.09 × 10^3^ km^2^ and 481.16 × 10^3^ km^2^, respectively. Given these findings, the value addition and market competitiveness of agricultural products can be enhanced through the development of regionally distinctive grain brands.

MLYP is the most suitable region for rice cultivation, with the largest actual and suitable planting areas, reaching 101.07 × 10^3^ km^2^ and 579.43 × 10^3^ km^2^, respectively. However, the energy and nutrient supply from grain crops in MLYP remains low. In response, greater investment is needed in the research and development of nutrient-rich rice varieties. Based on soil nutrient availability and rice growth requirements, precision fertilization and water-saving irrigation technologies should be implemented to enhance rice growth and optimize nutrient uptake. Additionally, with well-established supporting technologies and standardized operations, regionally appropriate rice-fish farming systems can be developed to enhance the ecological and economic benefits of paddy fields, boost the nutritional yield of aquatic products, and achieve a balance between the nutritional and sensory quality of rice [75].

YGP is the most suitable region for tuber cultivation, with actual and suitable planting areas reaching 59.00 × 10^3^ km^2^ and 336.58 × 10^3^ km^2^, respectively. Currently, this region has developed into a high-quality seed potato production base in southern China and plays a key role in supplying seed potatoes to countries and regions in South Asia and Southeast Asia. Therefore, leveraging the plateau’s natural conditions and developing a high-value seed potato industry is of great significance in encouraging farmer participation and advancing rural revitalization efforts [76]. Additionally, benefiting from its natural resource endowment, YGP exhibits high crop diversity in both actual and suitable conditions, with SDI value of 0.62 and 0.65, respectively, indicating a minimal gap between actual and suitable conditions. In this regard, a rotational cropping strategy involving different tubers, such as the “winter potato-summer sweet potato” rotation model, as well as cereals, legumes, green manure and other crops can be implemented to enhance grain crop diversity, thereby improving cropland use efficiency and comprehensive grain production capacity.

QTP is a region with the lowest nutrient value in grain crops, and the increase in nutrient supply between the suitable and actual condition is the smallest. In addition, local grain production in the region fails to meet regional food consumption and storage demands [77]. In this regard, while aligning with the carrying capacity of natural resources and the environment, the region needs to enhance support for breeding nutrient-rich grain crops and cultivate or introduce crop varieties enrich with key nutrients. By implementing these strategies, the nutritional supply capacity of regional grain crops can be enhanced, a rapid response to emergencies and natural disasters can be ensured, and the fundamental dietary needs of residents can be met. At the same time, QTP should fully leverage the benefits of crop diversity resulting from natural adaptation (with an SDI value 0.42 higher than the actual condition). It is essential to optimize crop distribution based on local ecological conditions and foster crop diversity while maintaining ecological balance. Additionally, the Comprehensive Development and Improvement Project of valleys on the Qinghai–Tibet Plateau should be advanced to enhance cropland quality. Furthermore, natural adaptation-based agricultural production and management strategies should be adopted to transition the dominant grain cropping system from a wheat-rice model to a tuber-based system, offering farmers new opportunities for income generation.

SC is a region with low grain crop energy density, with energy supply under actual and suitable conditions reaching 1.69 × 10^8^ KJ per 10,000 people and 16.75 × 10^8^ KJ per 10,000 people, respectively. Moreover, crop diversity in this region is relatively low, with an SDI of only 0.58 under suitable conditions. This is primarily because SC, as a major grain-exporting region, faces a significant imbalance between grain production and demand, which remains unresolved. In response to these challenges, it is essential to leverage technological advancements and practical expertise gained through digital transformation in the SC grain industry. Enhancing digitalization will accelerate the development of high-quality productivity in the grain sector and foster the sustainable and high-quality growth of grain production in this region.

In summary, this study incorporates nutritional security as a key indicator for assessing modifications in the grain crop planting pattern. It develops a nutrition-oriented approach to agriculture and serves as a decision-making framework for integrating nutrition and health objectives into food production restructuring strategies. Our findings are crucial for optimizing grain crop distribution, guiding agricultural practices and improving human dietary health.

### 4.3. Limitations and Future Directions

Although this study provides valuable insights into optimizing the planting strategies of regional grain crops to achieve stable food security, some limitations remain. Firstly, the analysis employed bioclimatic variables averaged for 1970–2000 to represent baseline conditions. Future research should incorporate updated datasets for 2000–2024 and projected scenarios for the future (e.g., SSP-RCP scenarios) to better capture potential shifts in crop suitability under anthropogenic climate change and to support more dynamic adaptation strategies. Secondly, crop planting data for wheat, maize, rice, soybean, potato, sweet potato and cassava were obtained from the SPAM database instead of field survey point data. Future studies could utilize higher-resolution or field-verified datasets to enhance spatial accuracy and model reliability. Thirdly, this study primarily focused on natural environmental variables in determining suitable planting areas. Integrating socio-economic and cultural dimensions—such as market demand, policy frameworks, and traditional dietary preferences—would provide a more holistic foundation for crop planning and decision-making. Meanwhile, the MaxEnt model results reflect only the natural site potential. Actual yields are shaped by varietal progress, i.e., genetic efficiency. In China, varietal progress (e.g., hybridization of high-yielding maize and rice) is responsible for the yield increase over the past decades [78,79]. The latest varieties are crucial in overcoming environmental constraints (e.g., drought and disease resistance) and allow for high yields to be achieved in regions that are only moderately suitable naturally. The subsequent work could combine naturally adapted planting patterns with appropriate, new varieties (genetic progress), which will be the optimal strategy for maximizing food production and nutrient supply. Furthermore, this study centered on production-side effectiveness and did not quantitatively assess grain losses or value reallocation during downstream stages such as processing and feed conversion. While this upstream focus estimates potential rather than realized dietary supply, it remains essential for identifying key production constraints and opportunities. Future research should adopt a full-chain analytical framework to more accurately depict the nutritional flow from field to table.

## 5. Conclusions

Theoretically, this study addressed a critical and timely issue at the intersection of food security, climate change, and nutrition supply in China, proposing region-specific strategies and providing a scientific basis for nutrition-oriented agricultural policy. Methodologically, it advances suitability modeling by incorporating topographic and soil variables into the MaxEnt framework alongside bioclimatic factors, enabling a more comprehensive evaluation of environmental constraints. Furthermore, by extending the assessment to planting diversity, production, and nutrient supply under the actual and naturally suitable conditions, this study offers a multidimensional perspective on optimizing crop systems for sustainable agricultural development. Our findings revealed distinct regional patterns in crop suitability. Wheat and maize are most suited to northern China, rice and tuber crops to southern regions, and soybean performs optimally in the northeast. In most regions, significant discrepancies exist between actual and suitable planting patterns, actual planting areas largely contained within potential suitable areas. Among alternative structures, maize-soybean and maize-based planting structures better facilitate natural adaptation and nutritional balance of the crop planting pattern (e.g., in Inner Mongolia and Heilongjiang), while rice-based structure show weaker alignment (e.g., in Shanghai). Future research should integrate updated climate scenarios, socio-economic dynamics, and full-chain food system analyses to guide adaptive and data-driven agricultural transformation.

## Figures and Tables

**Table 2:** The AUC values obtained after the suitability evaluation of 7 types of grain crops by MaxEnt model (data are means standing deviation (*n* = 10)).

Crop Type	Based on All Environmental Variables	Based on Major Environmental Variables
Wheat	0.9259 ± 0.0064	0.9172 ± 0.0071
Maize	0.8534 ± 0.0063	0.8425 ± 0.0066
Rice	0.9177 ± 0.0055	0.9056 ± 0.0057
Soybean	0.9354 ± 0.0075	0.9268 ± 0.0072
Potato	0.9370 ± 0.0108	0.9225 ± 0.0120
Sweet potato	0.9627 ± 0.0099	0.9498 ± 0.0083
Cassava	0.9932 ± 0.0032	0.9892 ± 0.0031

## Data Availability

The original contributions presented in the study are included in the article, further inquiries can be directed to the corresponding author.

## Supplementary Materials

The following supporting information can be downloaded at: https://www.mdpi.com/article/10.3390/foods14223870/s1, Figure S1: Distribution of planting sample points for wheat (a), maize (b), rice (c), soybean (d), and tuber (e) crops, Figure S2: Correlation analysis of the major environmental variables related to the planting suitability of wheat (a), maize (b), rice (c), soybean (d), potato (e), sweet potato (f), and cassava (g), Figure S3: The AUC values obtained in the models for wheat (a), maize (b), rice (c), soybean (d), potato (e), sweet potato (f), and cassava (g). Data are means ± standing deviation (*n* = 10), Figure S4: Actual planting patterns of wheat (a), maize (b), rice (c), soybean (d), and tuber (e) in China, Figure S5: The distribution map of the production of wheat (a), maize (b), rice (c), soybean (d) and tuber (e) in China under the actual condition, Figure S6: The distribution map of energy (a), protein (b), fat (c), and carbohydrate (d) contents provided by major grain crops in China under the suitable condition (rice as the standard food), Table S1: Environment variables used in the MaxEnt model and their descriptions, Table S2: Major environmental variables for evaluating wheat planting suitability (before screening), Table S3: Major environmental variables for evaluating wheat planting suitability (after screening), Table S4: Major environmental variables for evaluating maize planting suitability (before screening), Table S5: Major environmental variables for evaluating maize planting suitability (after screening), Table S6: Major environmental variables for evaluating rice planting suitability (before screening), Table S7: Major environmental variables for evaluating rice planting suitability (after screening), Table S8: Major environmental variables for evaluating soybean planting suitability (before screening), Table S9: Major environmental variables for evaluating soybean planting suitability (after screening), Table S10: Major environmental variables for evaluating potato planting suitability (before screening), Table S11: Major environmental variables for evaluating potato planting suitability (after screening), Table S12: Major environmental variables for evaluating sweet potato planting suitability (before screening), Table S13: Major environmental variables for evaluating sweet potato planting suitability (after screening), Table S14: Major environmental variables for evaluating cassava planting suitability (before screening), Table S15: Major environmental variables for evaluating cassava planting suitability (after screening), Table S16: Food equivalent coefficient of grain crops, Table S17: Comparison of actual planting SDI and suitable planting SDI of grain crops by province, Table S18: The difference between the per capita nutrient content of grain crops under suitable condition and actual condition (rice as the standard food).

## Abbreviations

The following abbreviations are used in this manuscript:

NCP	Northeast China Plain
NAS	Northern Arid and Semiarid Region
HHHP	Huang-Huai-Hai Plain
LP	Loess Plateau
QTP	Qinghai–Tibet Plateau
SBS	Sichuan Basin and Surrounding Region
MLYP	Middle-Lower Yangtze Plain
YGP	Yunnan-Guizhou Plateau
SC	Southern China
SDI	The Simpson Diversity Index, measuring the planting diversity of regional grain crops
CHO	carbohydrate
AUC	Area Under the Curve
