# Supplementary material for: Assessing the Alignment Between Naturally Adaptive Grain Crop Planting Patterns and Staple Food Security in China
Source: Foods. 2025 Nov 12;14(22):3870. doi: 10.3390/foods14223870 (PMC12651675; doi:10.3390/foods14223870)
Supplement: Supplementary file 1 [file foods-14-03870-s001.zip › foods-3955186-supplementary.pdf]

Supplementary Material

Assessing the Alignment between Naturally Adaptive Grain Crop Planting Patterns and Staple Food Security in China

Zhang et al.

Figures

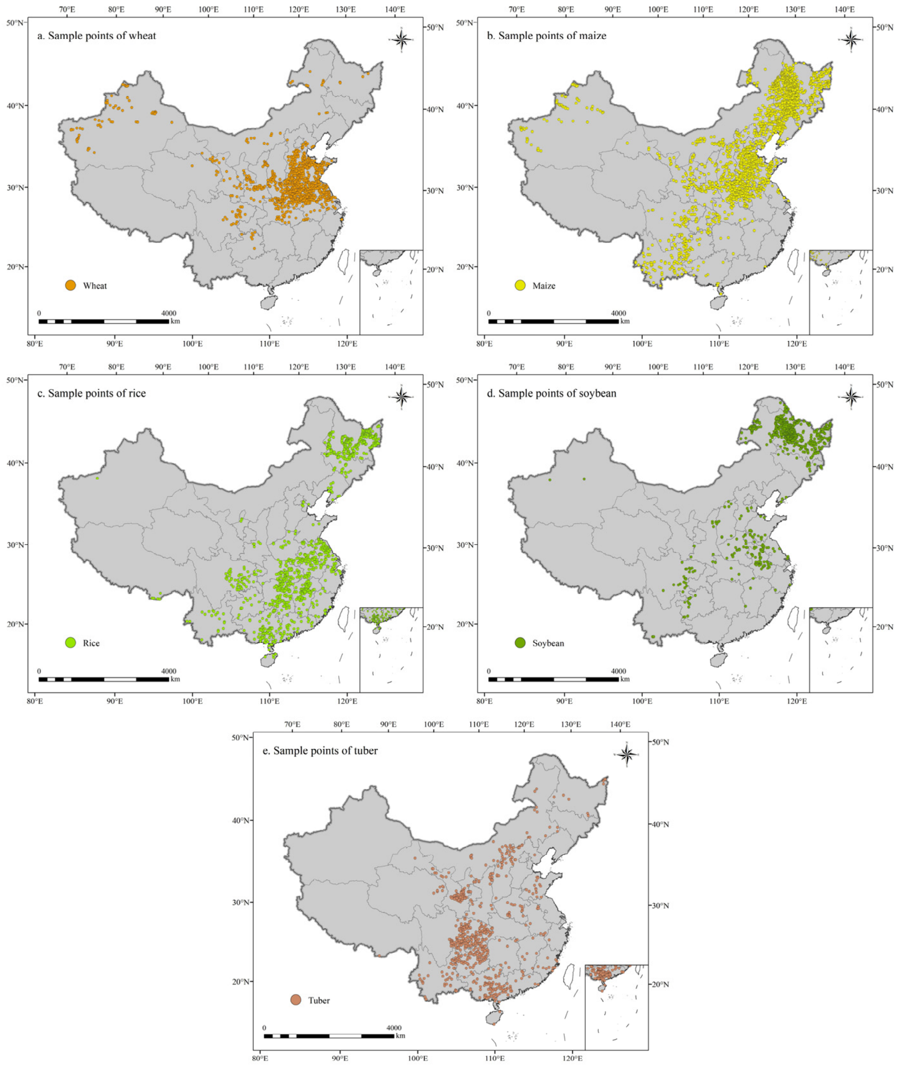

Figure.S1 Distribution of planting sample points for wheat (a), maize (b), rice (c), soybean (d), and tuber (e) crops.

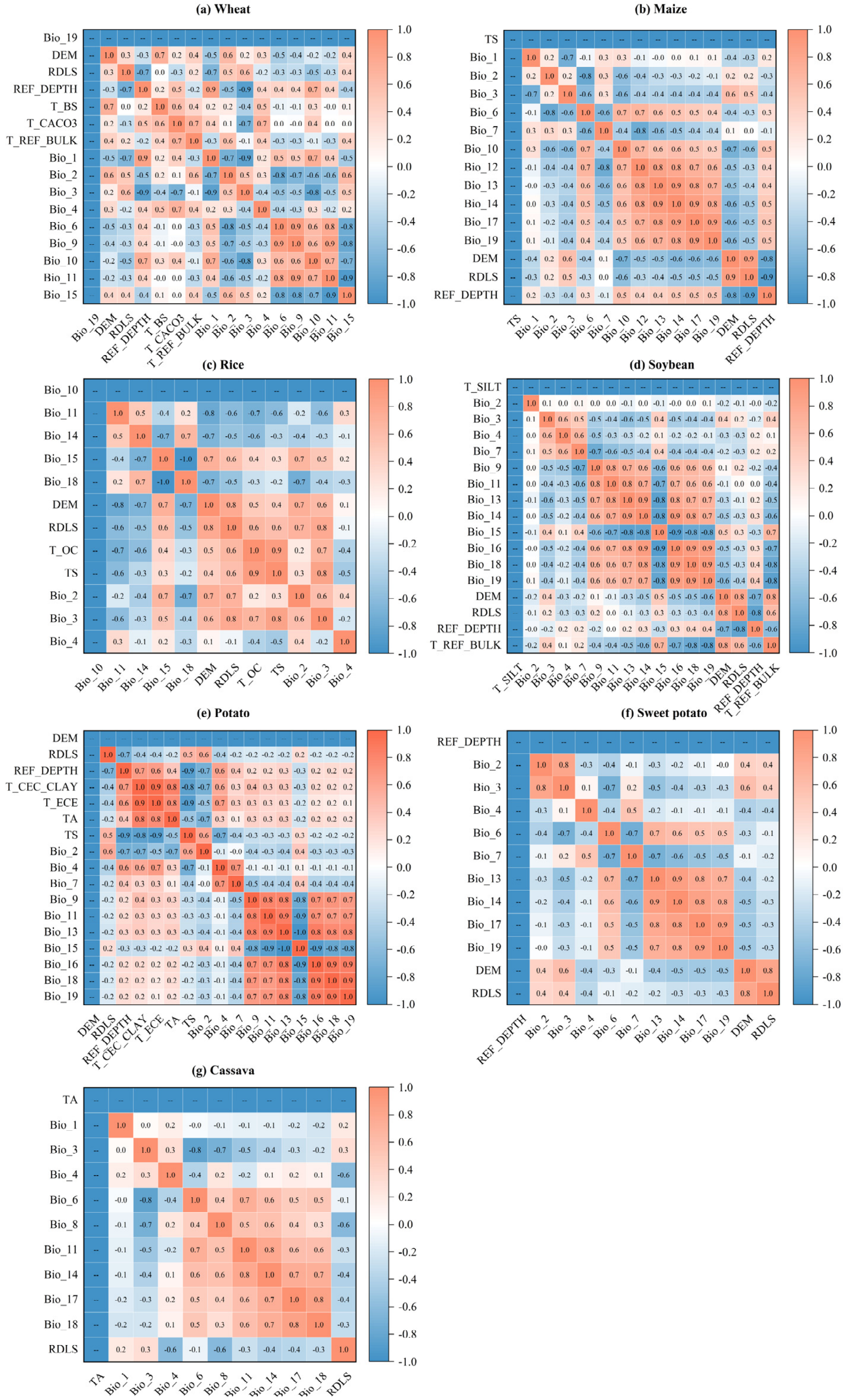

Figure.S2 Correlation analysis of the major environmental variables related to the planting suitability of wheat (a), maize (b), rice (c), soybean (d), potato (e), sweet potato (f), and cassava (g).

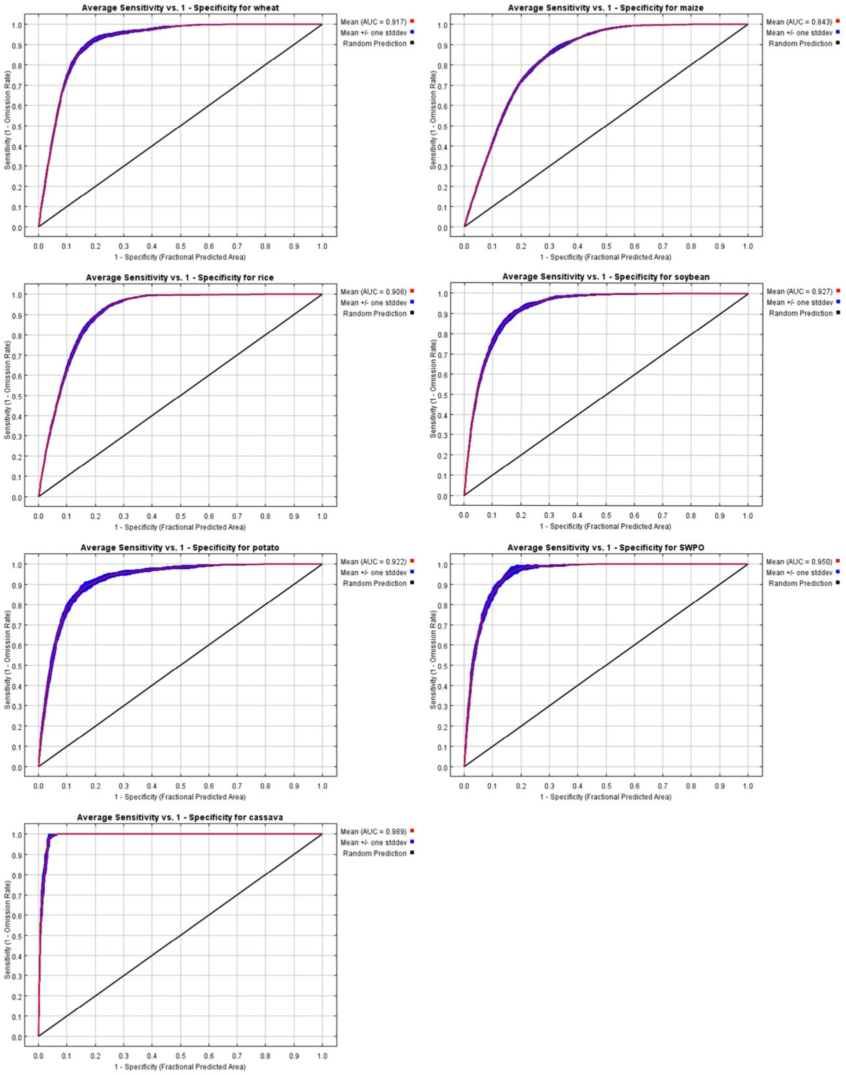

Figure.S3 The AUC values obtained in the models for wheat (a), maize (b), rice (c), soybean (d), potato (e), sweet potato (f), and cassava (g). Data are means ± standing deviation (n=10).

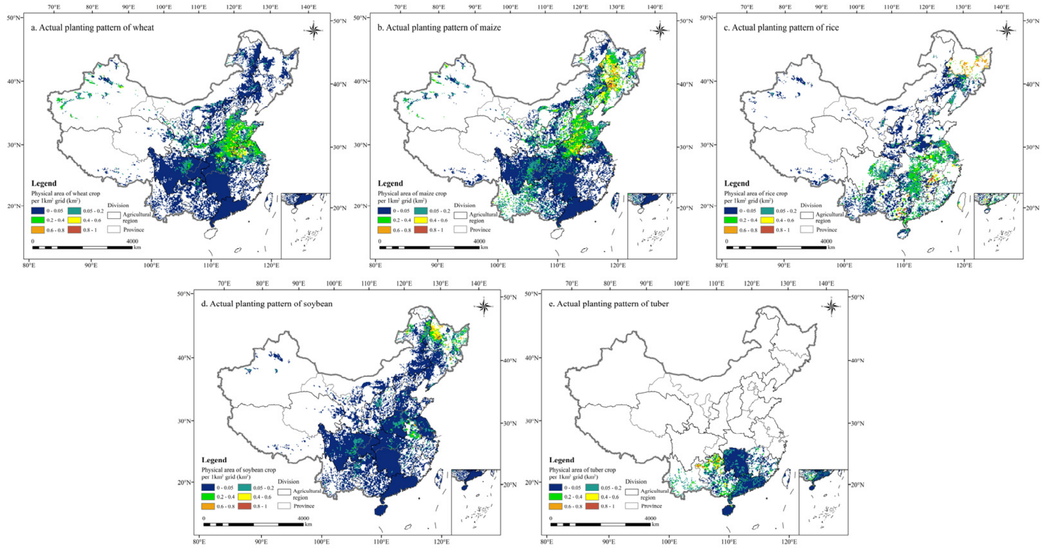

Figure.S4 Actual planting patterns of wheat (a) , maize (b), rice (c), soybean (d), and tuber (e) in China.

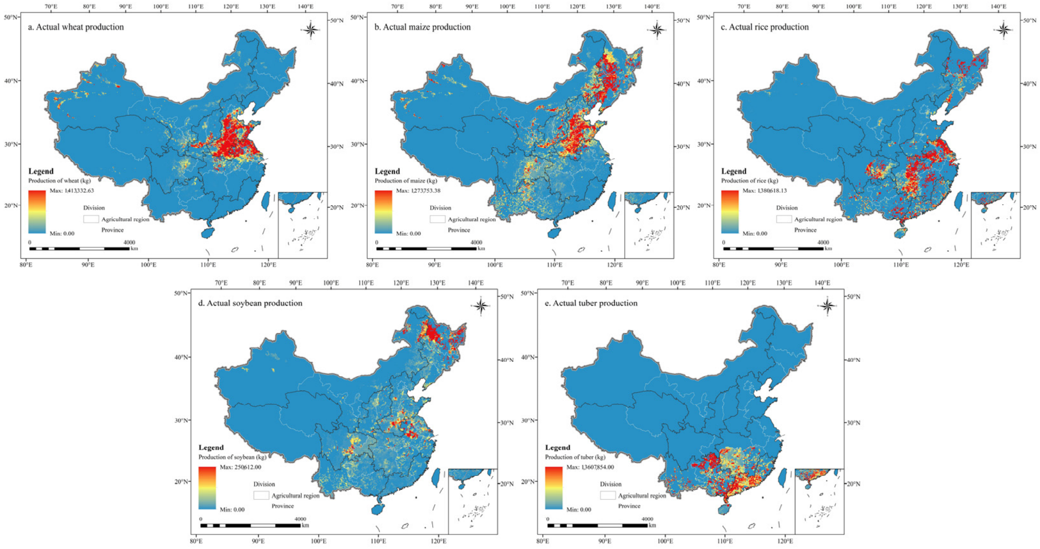

Figure.S5 The distribution map of the production of wheat (a), maize (b), rice (c), soybean (d) and tuber (e) in China under the actual condition.

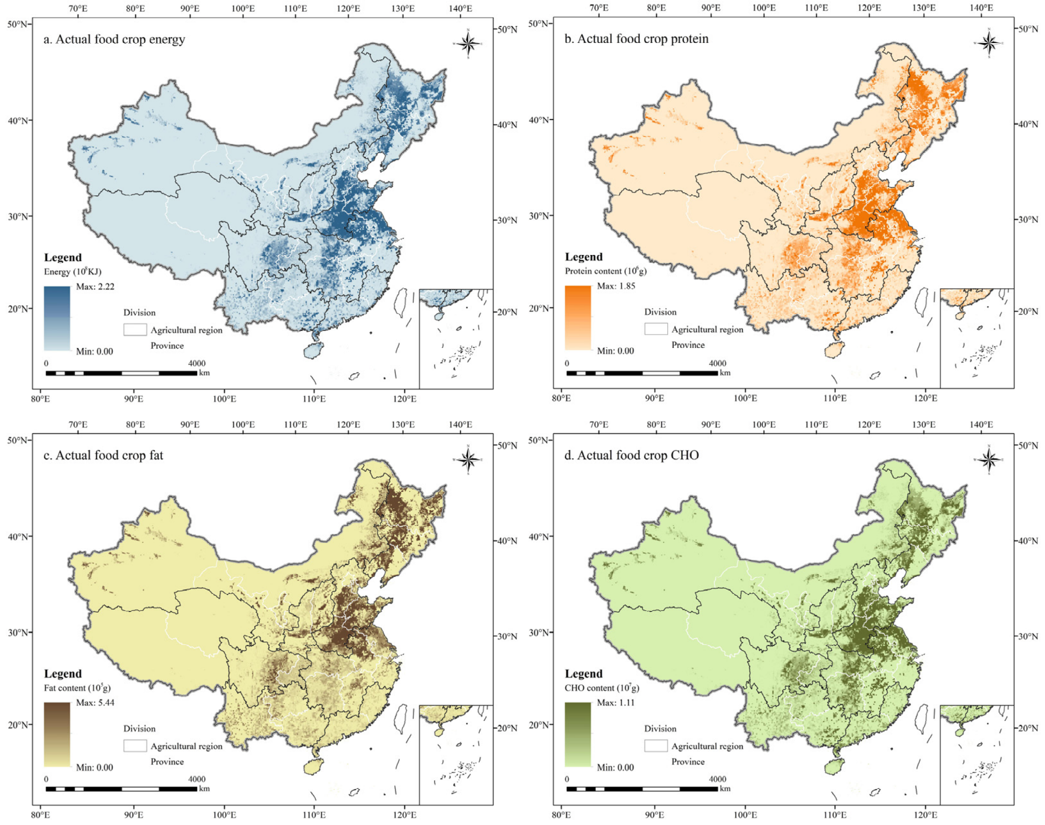

Figure.S6 The distribution map of energy (a), protein (b), fat (c), and carbohydrate (d) contents provided by major grain crops in China under the suitable condition (rice as the standard food).

Tables

Table.S1 Environment variables used in the MaxEnt model and their descriptions.

Type	Environment variable	Description	Unit
Bioclimate factors (19)	Bio1	Average annual temperature	℃
	Bio2	Average daily temperature difference	℃
	Bio3	Isothermal	-
	Bio4	Coefficient of seasonal variation of temperature	-
	Bio5	Maximum temperature in the hottest month	℃
	Bio6	Minimum temperature in the coldest month	℃
	Bio7	Annual difference in temperature	℃
	Bio8	Average temperature of the wettest quarter	℃
	Bio9	Average temperature of the driest quarter	℃
	Bio10	Average temperature of the warmest quarter	℃
	Bio11	Average temperature of the coldest quarter	℃
	Bio12	Annual precipitation	mm
	Bio13	Precipitation in the wettest month	mm
	Bio14	Precipitation in the driest month	mm
	Bio15	Seasonal variations in precipitation	mm
	Bio16	Precipitation in the driest quarter	mm
	Bio17	Wettest quarterly precipitation	mm
	Bio18	Warmest quarter precipitation	mm
	Bio19	Coldest quarterly precipitation	mm
Topography factors (4)	DEM	Altitude	m
	TS	Terrain slope	°
	TA	Terrain aspect	azimuth
	RDLS	Relief amplitude	—
Soil factors (19)	REF_DEPTH	Reference soil depth	code
	DRAINAGE	Drainage level	code
	AWC_CLASS	Range of effective soil water content	code
	T_GRAVEL	Topsoil gravel content	%vol
	T_SAND	Topsoil sand content	%weight
	T_SILT	Topsoil silt content	%weight
	T_CLAY	Topsoil clay content	%weight

T_USDA_TEX _CLASS	Topsoil USDA Soil Texture Classification	name
T_REF_BULK _DENSITY	Reference bulk density of topsoil	Kg/dm^3
T_OC	Topsoil organic carbon content	%weight
T_PH_H2O	Topsoil pH	-log(H)$^+$
T_CEC_CLAY	Cation exchange capacity of topsoil clay layer soil	cmol/kg
T_CEC_SOIL	Topsoil cation exchange capacity	cmol/kg
T_BS	Topsoil subgrade saturation	%
T_TEB	Topsoil exchangeable salt base	cmol/kg
T_CACO3	Topsoil carbonate or lime content	%weight
T_CASO4	Topsoil sulphate content	%weight
T_ESP	Topsoil exchangeable sodium salt	%
T_ECE	Topsoil conductivity	Ds/m

Table.S2 Major environmental variables for evaluating wheat planting suitability (before screening).

Variable	Percent contribution	Permutation contribution
RDLS	44.7	10.2
Bio_19	9.2	5.7
Bio_4	8.4	9.6
T_CACO3	5.0	0.8
DEM	3.6	20.6
Bio_3	2.5	2.3
Bio_11	2.3	3.2
T_BS	2.2	0.8
Bio_6	2.1	8.5
Bio_2	1.9	3.3
REF_DEPTH	1.8	0.5
Bio_9	1.8	1.1
Bio_15	1.7	1.7
Bio_10	1.6	0.2
T_REF_BULK	1.3	0.4
Bio_1	1	1.1

Table.S3 Major environmental variables for evaluating wheat planting suitability (after screening).

Variable	Percent contribution	Permutation contribution
RDLS	50.2	25.9

Bio_11	14.7	18.7
Bio_19	12.6	18.5
T_CACO3	7.8	0.5
Bio_4	6.9	19.3
Bio_3	1.6	2.7
Bio_2	1.5	8.3
Bio_15	1.4	3.7
T_REF_BULK	1.1	0.2
T_BS	0.9	0.9
REF_DEPTH	0.7	0.9
Bio_10	0.5	0.6

Table.S4 Major environmental variables for evaluating maize planting suitability (before screening).

Variable	Percent contribution	Permutation contribution
RDLS	36.0	21.5
Bio_19	13.2	14.5
Bio_12	7.8	8.1
REF_DEPTH	7.2	1.4
TS	4.7	5.0
Bio_10	4.3	12.1
DEM	3.9	2.2
Bio_14	3.2	2.2
Bio_13	3.1	0.5
Bio_1	2.3	14.5
Bio_2	2.0	1.9
Bio_17	1.7	2.8
Bio_6	1.5	0.3
Bio_3	1.3	0.5
Bio_7	1.2	1.0

Table.S5 Major environmental variables for evaluating maize planting suitability (after screening).

Variable	Percent contribution	Permutation contribution
RDLS	37.5	15
Bio_19	17.5	18.5
Bio_12	14.6	16.1
Bio_10	9.2	30.5
REF_DEPTH	7.7	0.6
TS	6.1	7.9
Bio_2	3.2	5.4
Bio_1	2.3	4.1

Bio_3	1.4	0.8
Bio_7	0.6	0.9

Table.S6 Major environmental variables for evaluating rice planting suitability (before screening).

Variable	Percent contribution	Permutation contribution
RDLS	64.3	7.3
DEM	4.5	4.2
Bio_18	4.0	3.4
Bio_2	2.9	3.3
Bio_3	2.8	1.1
T_OC	2.3	1.2
Bio_11	2.1	2.3
Bio_4	2.1	1.2
TS	1.6	3.2
Bio_14	1.4	2.0
Bio_15	1.1	1.7
Bio_10	1.0	37.7

Table.S7 Major environmental variables for evaluating rice planting suitability (after screening).

Variable	Percent contribution	Permutation contribution
RDLS	66.3	41.3
Bio_18	11.6	22.3
Bio_4	4	4.8
TS	3.5	7.4
T_OC	3.2	1.1
Bio_3	2.9	2.5
Bio_15	2.9	2
Bio_11	2.4	11.8
Bio_14	1.9	3.2
Bio_2	1.4	3.7

Table.S8 Major environmental variables for evaluating soybean planting suitability (before screening)

Variable	Percent contribution	Permutation contribution
Bio_4	31.6	1.1
RDLS	23.5	6.7
DEM	6.3	4.6
Bio_13	5.4	2.7
Bio_9	4.9	2.6
Bio_18	4.1	13.1

REF_DEPTH	2.9	0.3
Bio_11	2.3	11
Bio_14	1.8	8.1
Bio_7	1.4	1.1
Bio_3	1.4	0.3
Bio_16	1.2	7.1
Bio_15	1.1	1.1
T_REF_BULK	1.1	0
Bio_2	1.1	0.4
T_SILT	1.0	0.6
Bio_19	1.0	0.5

Table.S9 Major environmental variables for evaluating soybean planting suitability (after screening)

Variable	Percent contribution	Permutation contribution
Bio_4	33.4	6.7
RDLS	28.7	49.3
Bio_13	14.8	16.8
Bio_9	6.4	8.7
Bio_3	4.9	0.8
Bio_14	4.3	13.1
REF_DEPTH	2.1	0.3
Bio_15	1.7	2.1
Bio_2	1.6	0.7
T_SILT	1.2	0.7
T_REF_BULK	0.9	0.6

Table.S10 Major environmental variables for evaluating potato planting suitability (before screening)

Variable	Percent contribution	Permutation contribution
DEM	16.9	10.4
Bio_13	14.8	14.9
RDLS	13.1	9.1
Bio_2	8.7	2.8
Bio_19	7.1	8.3
REF_DEPTH	4.6	0.8
Bio_4	4.3	0.4
Bio_11	4.3	0.1
Bio_16	3	2.3
T_ECE	2.3	1.3
Bio_18	2.2	1.3
TS	2.1	3.0

Bio_7	1.4	1.7
Bio_9	1.3	2.1
TA	1.1	1.1
Bio_15	1.1	3.3
T_CEC_CLAY	1.0	1.9

Table.S11 Major environmental variables for evaluating potato planting suitability (after screening)

Variable	Percent contribution	Permutation contribution
DEM	29.9	39.1
Bio_16	13.5	15.9
Bio_2	10.9	6.9
Bio_19	10.1	9.2
Bio_13	9.8	13.5
Bio_4	7.3	0.8
REF_DEPTH	5.1	0.4
Bio_11	4.9	3.1
TS	2.7	3.6
T_ECE	2.2	1.0
Bio_15	2.1	4.0
T_CEC_CLAY	0.9	2.0
TA	0.6	0.5

Table.S12 Major environmental variables for evaluating sweet potato planting suitability (before screening)

Variable	Percent contribution	Permutation contribution
Bio_14	39.8	16.1
Bio_2	16.6	3.0
Bio_13	7.4	15.7
Bio_3	7.1	5.4
Bio_17	3.6	4.8
Bio_6	3.5	5.9
RDLS	2.4	2.4
Bio_4	1.9	2.8
Bio_19	1.7	3.3
DEM	1.5	6.4
Bio_7	1.1	0.2
REF_DEPTH	1.0	6.3

Table.S13 Major environmental variables for evaluating sweet potato planting suitability (after screening)

Variable	Percent contribution	Permutation contribution
Bio_14	42.3	10.9
Bio_2	24.9	7.6
Bio_13	11.5	31.8
Bio_3	9	6
Bio_6	6.7	30.2
RDLS	3	3.7
Bio_4	1.6	8.4
REF_DEPTH	1.1	1.5

Table.S14 Major environmental variables for evaluating cassava planting suitability (before screening)

Variable	Percent contribution	Permutation contribution
Bio_17	36.9	0.2
Bio_1	25.4	24.8
Bio_3	5.9	4.2
Bio_18	4.3	1.7
Bio_14	3.7	1.2
RDLS	3.3	8.8
Bio_11	2.4	0.6
Bio_4	2.4	4.6
Bio_8	2.1	13.2
Bio_6	2.0	1.1
TA	1.0	1.5

Table.S15 Major environmental variables for evaluating cassava planting suitability (after screening)

Variable	Percent contribution	Permutation contribution
Bio_17	41.8	0.7
Bio_1	39.4	35.9
Bio_18	5.3	6.9
Bio_3	4.7	3.4
RDLS	3.6	18.8
Bio_8	3.1	10.9
Bio_4	1.1	22.5
TA	1.0	0.9

Table.S16 Food equivalent coefficient of grain crops

	Wheat	Maize	Rice	Soybean	Tuber
FEU	1.03	1.01	1.00	1.45	0.25

FEU'	Wheat	Maize	Rice	Soybean	Tuber
	1.07	1.01	1.00	1.51	0.06

Table.S17 Comparison of actual planting SDI and suitable planting SDI of grain crops by province

Province	SDI		Province	SDI	
	Actual	Suitable		Actual	Suitable
Beijing	0.40	0.64	Hubei	0.71	0.79
Tianjin	0.57	0.70	Hunan	0.54	0.62
Hebei	0.49	0.69	Guangdong	0.58	0.56
Shanxi	0.47	0.71	Guangxi	0.64	0.61
Inner Mongolia	0.60	0.69	Hainan	0.06	0.59
Liaoning	0.26	0.69	Chongqing	0.64	0.70
Jilin	0.36	0.68	Sichuan	0.69	0.74
Heilongjiang	0.66	0.68	Guizhou	0.61	0.69
Shanghai	0.42	0.63	Yunnan	0.60	0.64
Jiangsu	0.65	0.75	Tibet	0.24	0.72
Zhejiang	0.62	0.63	Shaanxi	0.62	0.73
Anhui	0.70	0.77	Gansu	0.60	0.70
Fujian	0.57	0.59	Qinghai	0.31	0.67
Jiangxi	0.39	0.46	Ningxia	0.56	0.68
Shandong	0.55	0.75	Xinjiang	0.54	0.61
Henan	0.61	0.76			

Table.S18 The difference between the per capita nutrient content of grain crops under suitable condition and actual condition (rice as the standard food)

Province	The difference between suitable and actual per 10,000 people nutrient content of food crops			
	energy (10^8KJ)	protein (10^6g)	fat (10^6g)	CHO (10^7g)
Beijing	14.24	10.65	3.41	7.18
Tianjin	16.60	8.53	2.47	5.21
Hebei	46.56	129.18	35.87	79.43
Shanxi	47.64	57.92	15.95	38.74
Inner Mongolia	343.51	314.65	127.98	178.55
Liaoning	72.37	109.62	38.45	68.57
Jilin	129.31	119.05	44.11	66.73
Heilongjiang	261.99	371.82	142.62	168.05
Shanghai	5.39	4.34	0.92	3.10
Jiangsu	37.58	115.02	26.76	72.43
Zhejiang	13.62	25.18	4.25	20.86
Anhui	49.80	103.39	22.81	70.40
Fujian	14.82	16.34	2.51	14.78
Jiangxi	29.93	35.63	4.95	32.51
Shandong	44.51	165.19	45.15	103.00
Henan	42.27	156.11	33.14	97.43

Hubei	48.59	91.70	19.12	64.95
Hunan	27.60	52.52	9.17	43.35
Guangdong	11.00	36.08	5.18	33.46
Guangxi	24.38	32.95	5.86	29.22
Hainan	19.35	5.16	0.91	4.69
Chongqing	25.64	27.09	7.65	18.77
Sichuan	28.83	85.32	26.73	53.89
Guizhou	24.98	30.27	8.72	22.25
Yunnan	30.47	41.26	14.07	33.67
Tibet	48.44	9.13	3.60	3.39
Shaanxi	52.70	71.90	19.48	48.56
Gansu	47.32	38.62	11.36	27.80
Qinghai	24.39	5.28	1.64	3.27
Ningxia	42.79	9.95	2.96	7.30
Xinjiang	137.61	114.48	34.13	83.89
